# Multiple Leader Candidate and Competitive Position Allocation for Robust Formation against Member Robot Faults 

**DOI:** 10.3390/s150510771

**Published:** 2015-05-06

**Authors:** Ji-Wook Kwon, Jin Hyo Kim, Jiwon Seo

**Affiliations:** 1 Yonsei Institute of Convergence Technology, Yonsei University, 85 Songdogwahak-ro, Incheon 406-840, Korea; E-Mails: bluemichael@yonsei.ac.kr (J.-W.K.); jinhyo.kim@yonsei.ac.kr (J.H.K.); 2 School of Integrated Technology, Yonsei University, 85 Songdogwahak-ro, Incheon 406-840, Korea

**Keywords:** formation control, robust formation, multiple leader candidate structure, competitive position allocation algorithm

## Abstract

This paper proposes a Multiple Leader Candidate (MLC) structure and a Competitive Position Allocation (CPA) algorithm which can be applicable for various applications including environmental sensing. Unlike previous formation structures such as virtual-leader and actual-leader structures with position allocation including a rigid allocation and an optimization based allocation, the formation employing the proposed MLC structure and CPA algorithm is robust against the fault (or disappearance) of the member robots and reduces the entire cost. In the MLC structure, a leader of the entire system is chosen among leader candidate robots. The CPA algorithm is the decentralized position allocation algorithm that assigns the robots to the vertex of the formation via the competition of the adjacent robots. The numerical simulations and experimental results are included to show the feasibility and the performance of the multiple robot system employing the proposed MLC structure and the CPA algorithm.

## 1. Introduction

Formation control of a multi-robot system has been a big challenge for the robot control society. Contrasting with a single robot system, since the robots in the formation are interconnected with others, higher level control techniques have been required. Nevertheless, the formation control has been expected to be employed in various applications such as manipulation of large objects [1,2], intelligent highway systems [3,4], surface vehicle formation [5], flight formation systems [6,7], formation of multiple spacecraft [8], and surveillance systems [9,10,11,12] because the multi-robot system can provide certain advantages including cost reduction, sensing performance improvement, and higher robustness against robot faults. In particular, the robotic-sensor networks [10,11,12] in the surveillance systems have been a focus of attention because they reduce the cost of the surveillance systems and increase the performance.

Many formation control algorithms have been studied based on various strategies including leader-follower algorithms [13,14,15,16,17], virtual structure methods [2], behavior based formation control algorithms [18,19], and potential field based formation control algorithms [20]. These formation control strategies can be largely divided into two strategies with respect to the type of leader of the entire system: (a) virtual-leader strategy and (b) actual-leader strategy.

First, under the virtual-leader strategy, all robots generate the virtual leader which is a simulated leader regarding a formation trajectory. The virtual structure methods are typical case of the virtual-leader strategy. To implement this strategy, all robots should have the formation trajectory, measure the global position, and share and synchronize all the information. These requirements provide the advantage of robustness against the fault or disappearance of member robots. However, the virtual-leader strategy should require a high cost and high performance system because all robots acquire the same information at the same time. 

On the other hand, under the actual-leader strategy, the leader is one of the member robots of the formation. The leader-follower formation algorithms are a typical case of the actual-leader strategy. To implement the actual-leader strategy, the leader has the trajectory of the entire formation and measures the global position, and the others (*i.e.*, the followers) maintain the formation using the relative information. This drives the cost reduction since all robots do not synchronize their own information and all followers do not require the global position. However, if the leader has disappeared or has a fault, the entire system cannot be maintained since the global position and the trajectory information are not acquired. 

To achieve robustness against faults of the leader, leaderless and leader-formation control based on consensus algorithms have been developed [21,22,23,24]. These leaderless and leader-formation control algorithms employ multiple leaders and use the relative information between robots. The consensus-based formation algorithms are more robust against leader faults than the actual-leader strategy and require lower cost than the virtual-leader strategy. However, the formation with the consensus-based formation control suffers from big movement load, difficulty of rotation of the formation, and weak robustness against the faults of followers due to the rigid-allocation, and whereas the rigid-allocation results in easy maintenance of the formation, the multi-robot system employing the rigid-allocation is weak in terms of member robot faults. Moreover, the rigid-allocation increases the movement load of the entire system because it does not regard the moving distances and loads of all robots.

In addition, leader election algorithms have been researched, such that the leader is selected among the members of the mobile sensor networks (see [25,26] and references therein). In [25,26], it is mentioned that robustness against leader faults can be achieved because the missing leader can be replaced by a new leader elected from a proposed leader list which is a set of leader candidates. However, in [25,26], the relationship between the candidates and the leader position is rigid, and also, the followers are assigned to the formation using rigid allocation. Whereas these leader election algorithms achieve robustness against the fault of the leader, they do not reduce the moving cost of the entire system.

Finally, a formation control strategy based on the on-line position allocation was proposed in [27,28]. All robots are allocated to the vertices of the formation with respect to some moving distance of the entire system. However, to optimize the moving distance of the entire system, all states of all robots should be synchronized. Whereas the on-line allocation and minimized moving distance are achieved, the synchronization of all states of all robots increases the cost of the entire system.

Thus, we propose a Multiple Leader Candidate (MLC) strategy and a Competitive Position Allocation (CPA) algorithm. First, the MLC strategy employs multiple candidates for the leader position. One of the candidate robots occupies the leader position via the CPA algorithm. The winner becomes the leader and the others become the followers. If the leader disappears or breaks down, all candidate robots moving as the followers compete again for the leader position to replace the missing leader. Second, the proposed CPA algorithm is the distributed online allocation algorithm. In this paper, the position allocation of the followers and the competition of the leader candidate robots are done by the CPA algorithm. Each follower is allocated the closest vertex of the formation. If there are other followers with the same vertex, they compete for the vertex. The winner occupies the vertex, and the losers find other vertices. Note that, in the case of the MLC, the vertex is the leader position.

Using the MLC strategy and the CPA algorithm, the following contributions are achieved: the formation of multiple robots is robust against the fault of the leader and the followers. Unlike the previous formation control strategies based on rigid-allocation, such as the actual-leader strategy and consensus-based formation control, the entire system can be maintained despite the faults of the leader or the followers because the other robots replace the broken or missing robots. A formation of multiple robots requires low cost to build the entire system. In contrast with the formation control strategies using all information of all robots, such as the virtual-leader strategy and the optimization-based position allocation algorithm, the cost of the entire system is reduced since the local information is used for the on-line position allocation.

This paper is organized as follows: in Section 2, the multi-robot system considered in this paper is explained. The CPA algorithm and the MLC strategy are explained in Section 3. To demonstrate the usefulness of the proposed control algorithms, numerical simulations and experimental results are presented in Section 4, and, finally, in Section 5, the conclusions of the study are given.

## 2. Multiple Robot System Construction 

**Figure 1:**
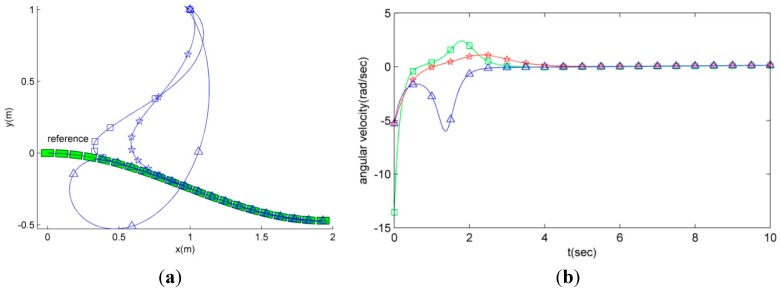
The comparison of the movement loads of the proposed feedback linearization algorithm and the others (☆: the proposed algorithm, ∆: the nonlinear controller, and □: PID controller). (**a**) The route of the mobile robots; (**b**) the angular velocities.

This section describes a kinematic model of the robots with the non-holonomic constraint and the relationship between them. The robot considered in this paper is described as follows:

(1) $$\left[\begin{array}{c} x_{i}\\ y_{i}\\ \theta_{i} \end{array}\right]=\left[\begin{array}{cc} \cos\theta_{i} & 0\\ \sin\theta_{i} & 0\\ 0 & 1 \end{array}\right]\left[\begin{array}{c} v_{i}\\ \omega_{i} \end{array}\right]$$

where (*x*, *y*) is the position of the robot, θ is an orientation, *v* is a linear velocity, ω is an angular velocity, and the subscript *i* is the index number. The kinematic model, non-holonomic constraint, and the motions of the robots influence the performance of the multi-robot system because the relationship between mobile robots, a communication graph, and cost of the entire system are changed due to the movements of the robots. Therefore, the control law of the mobile robot with the kinematics in Equation (1) should be considered. To control all robots, we employ the motion control law based on the Feedback Linearization (FL) in [29,30] (see [30] for details of the control law and to check the stability). This FL-based motion control law can reduce the movement load of each robot. Figure 1 shows the performance of the FL control law.

Figure 1a compares the three routes of the robots using the FL-based motion controller, PID controller, and the nonlinear control algorithm in [31]. As can be seen in Figure 1a, the route of the FL control law is simpler and shorter than the others. Also, we can see a smaller peak of the angular velocity of the robot with the FL-based motion control than the other control laws in Figure 1b. Thus, when the situations of the formation change and the leader disappearances emerge, it is possible that the FL based motion control algorithm reduces the movement load of each robot. To describe the formation, we employ *l*-ψ description with respect to the leader position in [32]. We describe the formation as a set of vertices as follows:

(2) $$G=\left\{g_{j}\left(x_{j}^{d},y_{j}^{d}\right)|j=1,...,n\right\}\;x_{j}^{d}=x_{L}+L_{j}\cos\left(\theta_{L}+\psi_{j}\right),y_{j}^{d}=y_{L}+L_{j}\sin\left(\theta_{L}+\psi_{j}\right)$$

where $\left(x_{j}^{d},y_{j}^{d}\right)$ is the position of the *j*th vertex *g_j_*, (*x_L_*, *y_L_*) is the position of the leader, θ*_L_* is the orientation, *n* is the number of the vertices of the formation, *L_j_* is a distance of the *j*th vertex from the leader, and ψ*_j_* is an angular position regarding the orientation of the leader. When *L* and ψ are defined as a set of *L_j_* and ψ*_j_*, the vertex set of the formation is revised as:

(3) $$G=\left\{L,\psi \right\}\;L=\left\{L_{j}|j=1,...,n\right\},\psi =\left\{\psi_{j}|j=1,...,n\right\}$$

**Figure 2:**
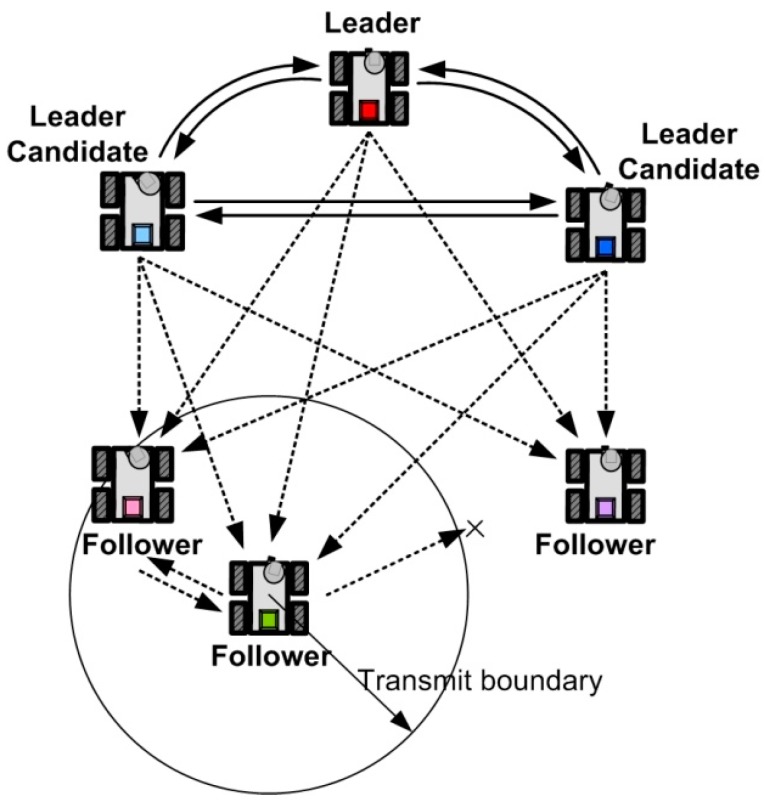
The combined communication structure.

To implement the proposed MLC strategy and CPA algorithm, we construct the combined communication structure between the actual-leader and the virtual-leader structures. This proposed communication structure is depicted in Figure 2.

As can be seen in Figure 2, the communication between the leader and all the leader candidates (LCs) employs the virtual-leader structure where their information is shared and synchronized to monitor the fault of the leader. That is, the LCs and the leader have a complete network topology. On the other hand, the information of the leader and LCs is broadcast to the followers via the actual-leader structure. Because of this broadcasting information of the leader and LCs, the followers can acquire the leader information. Finally, the followers communicate with other followers in their limited communication range to reduce the energy consumption. In spite of the complete network topology of the LCs, the communication network of followers is not fixed. It can be noted here that the communication graph of the proposed combined communication structure has a switched network topology.

## 3. Multiple Leader Candidate Structure with Competitive Position Allocation

This section shows the MLC structure with the multiple leader candidates and CPA algorithm based on competition using local information. The proposed MLC structure and CPA algorithm are designed to be implemented in the multi-robot system using a behavior-based control algorithm [19,33]. Whereas it is difficult to show the feasibility and stability of the behavior-based control laws using the mathematical tools [32], the behavior-based control scheme is good to implementation in actual multi-robot systems in the real world.

### 3.1. Multiple Leader Candidate Structure

**Figure 3:**
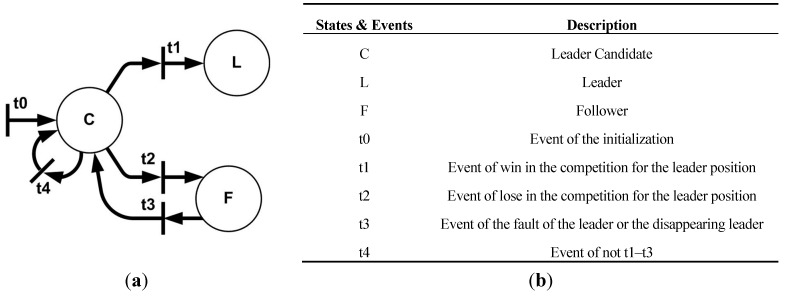
The task assignment of LC. (**a**) The task assignment process; (**b**) States and events in (a).

To achieve the robustness against the fault of the leader, we propose the MLC structure. The winner of the competition of LCs for the leader position becomes the leader of the entire system. By the proposed MLC structure, all robots are allowed the following three tasks: (a) the leader; (b) the follower; and (c) the LC. First, the leader is selected from the LCs and leads the entire system along the trajectory of the formation. Second, the followers only track vertices of the formation with respect to the leader without regard to the global information such as the position and trajectory of the entire system. If there is no leader, followers trace the vertices with respect to the closest LC. Finally, the LC becomes the leader or follower. If there is no leader, the LCs compete to occupy the leader position. The task assignment of LC is shown in Figure 3.

As can be seen in Figure 3, a winner of the competition becomes the leader and the losers become the followers. In Figure 3, in the initial condition, all the LC robots are assigned task C, and then, by the events, t1 and t2, the robots become leader (L) and follower (F), respectively. If t3 occurs, they revert to task C to replace the empty leader position. Here, unlike the native followers, the LCs assigned to F monitor the leader to replace the leader in the case of breakdown. Accordingly, in the proposed MLC structure, LC is the important task since the leader is selected among LC robots, and they can replace the broken (or lost) leader.

Note that the MLC structure is scalable since all LCs share their information in virtue of the virtual-leader structure. If the number of the LCs is chosen with respect to the performance of the communication devices, the MLC structure is maintained when the number of the robots increases.

### 3.2. Competitive Position Allocation Algorithm

**Figure 4:**
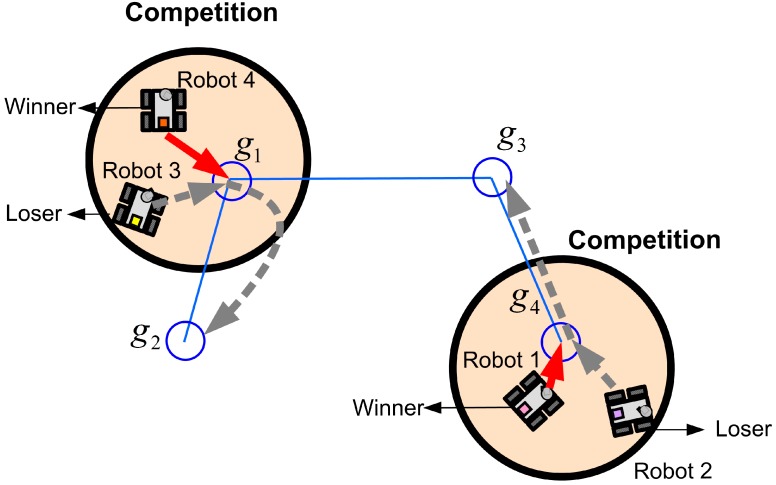
The competition of the followers for their vertices.

The proposed CPA algorithm is the distributed position allocation method, such that the followers are allocated to the vertices of the formation via the competition using the local information instead of the optimization using all the information of entire system. The proposed CPA algorithm is applied to two cases: the competition for the leader position and the position allocation of followers. First, in the case of the leader selection, all LCs compete for the leader position. It is possible that all LCs monitor the leader and replace the missing leader since the virtual-leader strategy connects all LCs. By the task assignment in Figure 3, if the leader is lost or breaks down, the LCs compete for the leader position again. Second, if the CPA algorithm is applied to the position allocation of followers, the followers compete for vertices of the formation. By the CPA algorithm, each follower tracks the closest vertex. If there are other followers sharing the same vertex, they compete to occupy the vertex. Figure 4 shows this position allocation of the followers.

Figure 4 shows the situation in which four robots are allocated to four vertices by the proposed CPA algorithm. As can be seen in Figure 4, the winner of the competition occupies the selected closest vertex, and the loser selects again the next closest vertex in the set of goals, *G*, except the losing vertex. When this process is repeated, all followers are allocated to all vertices of the formation. For example, because Robot 1 and Robot 2 share g4 and they communicate with each other, the competition for *g*4 emerges. When the distances between robots and *g*4 are compared, Robot 1, the closest robot to *g*4, wins the competition and occupies *g*4. On the other hand, Robot 2, which is the loser of the competition, finds the next closest vertex, and then Robot 2 moves toward *g*3. The details of this position allocation algorithm based on the competition are clearly described in Algorithm 1.

In Algorithm 1, the taboo list presents the available goals in *G*. As in Algorithm 1, CPA algorithm has three parts: (a) finding the closest goal; (b) evaluating the selected goal; and (c) evaluating *T*. Since the CPA algorithm is based on the local information acquired in the limited communication range, it should be required to evaluate the feasibility of the selected vertex and the taboo list. First, a follower selects the closest vertex among unmarked vertices in the taboo list. Then the selected vertex is evaluated based on the existence of closer followers to the vertex. If the follower is the closest one, then the selected vertex is occupied by the follower. However, if any other robot is closer than the follower, the selected goal, *T*(*j*), is marked and the first process is repeated. In addition, if there is any robot with the same distance and smaller ID, the selected goal, *T*(*j*), is marked and the first process is repeated. Finally, because the CPA algorithm is based on the local information, all the goals in the taboo list might be marked without occupying the goal. Thus, we include the fifth process evaluating the taboo list.

**Algorithm 1** The competition of the followers for their vertices.

1. Find the closest goal in goal set.
2. Check that there are other robots in the communication range, which are closer to the selected goal.
3. If there are no closer robots,
           then, occupy the selected goal.
4. else if there is any closer robot
          then, mark taboo list, *T*(*j*) = 1 and return to the process 1 except the marked goal in *T*.
5. else if there is any robot with same distance
          If there is a robot with a smaller ID
               Then, mark taboo list, *T*(*j*) = 1 and return to the process 1 except the marked goal in *T*.
          else
                occupy the selected goal.
6. If the robot does not occupy any goal but all goals in *T* are marked
         then set the *T* as zero and return to the process 1.


*Remark 1*: Considering the communication model in Figure 2 and the proposed CPA algorithm in Algorithm 1, the competition of the adjacent followers occurs within a short communication boundary. Each follower cannot know the existence of the other followers sharing the same vertex if they are outside the communication boundary.

*Remark 2*: The followers employing the proposed CPA algorithm do not regard the entire system, but the adjacent robots within the communication boundary. The followers just receive the leader information from the leader and do not send information to the leader. Thus, the CPA algorithm is scalable and independent of the number of the robots.

## 4. Results

### 4.1. Simulation Results

To show the feasibility and the performance of the proposed MLC structure and CPA algorithm, three scenarios of the leader disappearing, formation changing situations, and large multi-robot system with 100 robots are included. Four mobile robots which have the kinematic model in Equation (1) and the FL motion control law are considered. The initial conditions of the robots are given as follows: 1st robot: (−2, 1, 0), 2nd robot: (2, 1, 0), 3rd robot: (−2, −1, 0), and 4th robot: (−2, −3, 0). Also, the initial condition and the velocities of the trajectory of the formation are (0, 0, −π) and {*v_r_* = 1.5 m/s, ω*_r_* = −0.2 tanh (0.1 × (*t* − 15)) rad/s}, respectively. Two formations are utilized: *G*1{*L*1{0, 2, 2, 2}, ψ1{0, −π/3, π/3, π}} and *G*2{*L*2{0, 3, 3, 3}, ψ2{0, 0, 2π/3, −2π/3}}, with the first and second robots as LCs. It can be assumed that the communication range of the followers (the 3th and the 4th robots) is 1 m.

**Figure 5:**
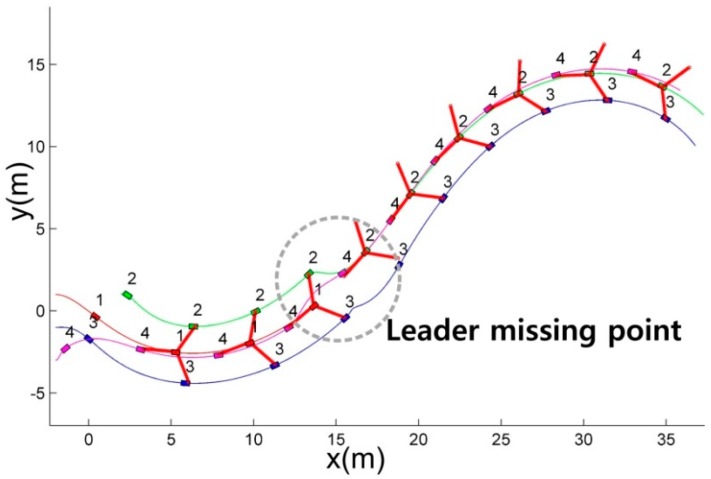
The route of the robots in the leader disappearing situation.

Scenario 1 shows the leader disappearing situation where the robots maintain *G*1, and the leader is lost at *t* = 10 s. Figure 5 shows the routes of the robots maintaining *G*1.

**Figure 6:**
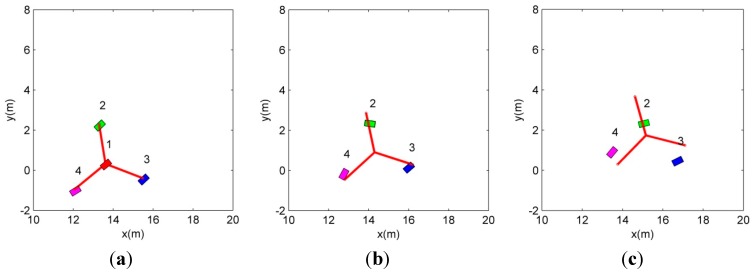

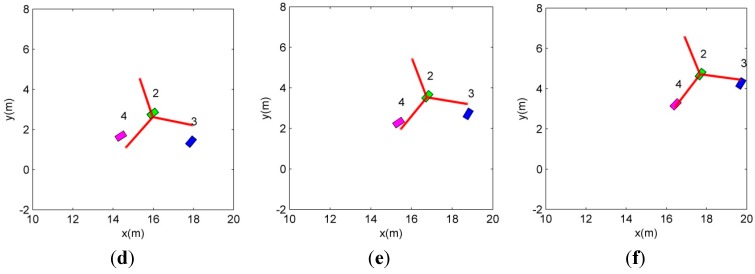
The details of the replacement of the leader.

At the start, the first robot becomes the leader since it is the winner of the competition for the leader position. As depicted in the dotted area in Figure 5, when the leader (first robot) is lost, the other LC (the second robot) occupies the leader position. To detail the replacement of the lost leader in the dotted region in Figure 5, Figure 6 shows the snapshots. The snapshots in Figure 6 show that the other LC (the second robot) replaces the lost leader (the first robot), and the followers (the third and fourth robots) reconstruct the formation with respect to the replaced leader (the second robot).

**Figure 7:**
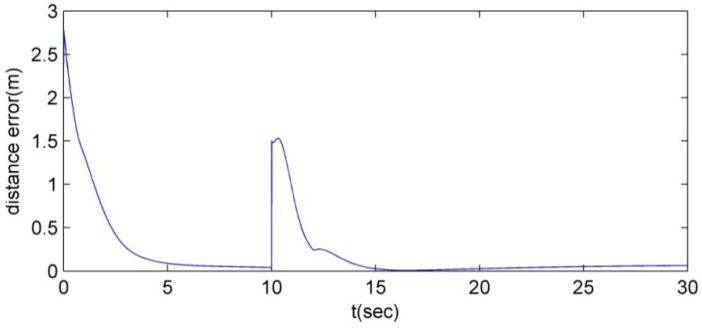
The competition of the followers for their vertices.

In addition, to show the performance of the motion control and the position allocation, Figure 7 shows the mean distance error of the four robots.

In Figure 7, since the robots are allocated and track the vertices before the leader disappearing situation, the distance errors between the robots and the vertices are bounded. When the leader is lost, the distance error of the formation increases because the vertices are regenerated with respect to the replaced leader. By the proposed CPA algorithm, the followers are reassigned to the new vertices. After the reassignment of the followers, the distance errors converge to zero and are bounded.

**Figure 8:**
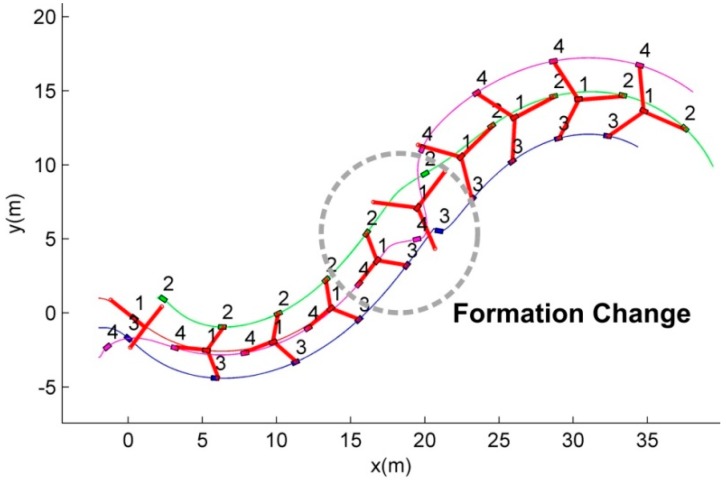
The route of the robots in the formation changing situation.

**Figure 9:**
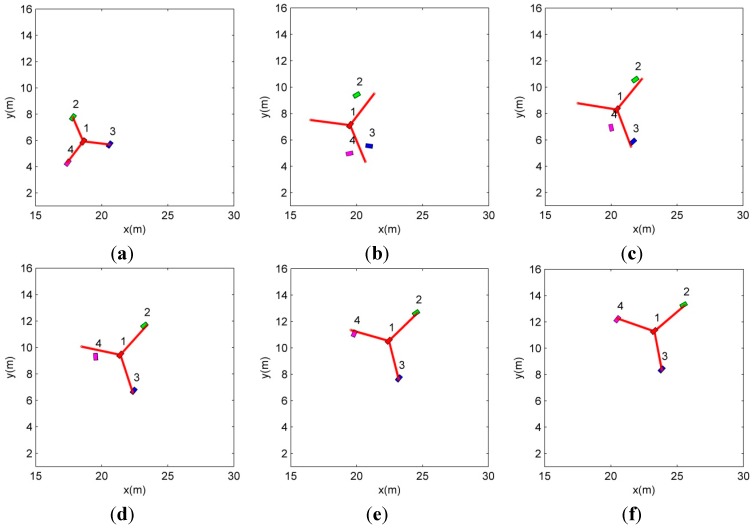
The movements of the member robots at the moment of the formation change.

Scenario 2 includes the formation change situation where the formation is changed from *G*1 to *G*2 at *t* = 15 s. Figure 8 shows the routes of the robots maintaining the given formations, G1 and G2. As can be seen in Figure 8, the robots construct and maintain the formations, *G*1 and *G*2, without the rigid allocation in the situation of the formation change as shown in the dotted region. To detail the reorganization of the formation in Figure 8, Figure 9 shows the snapshots of the movement of the robots.

The snapshots in Figure 9 show that the followers are allocated to the new vertices of the new formation (*G*2) by the proposed CPA algorithm. The robots maintaining *G*1 in Figure 9a are reallocated in the new formation *G*2 as in Figure 9b–f. In this scenario, when the formation is changed, all the robots except the leader (the first robot) compete for the new vertices of *G*2 as presented in Figure 9.

It can be noted that the third and fourth robots are allocated to the same vertex because they cannot detect each other due to the limited communication range as mentioned in Remark 1. When they are in the communication range, they compete for the selected vertex. In Figure 9b,c, the fourth robot loses the competition, thus it moves to the other vertex.

**Figure 10:**
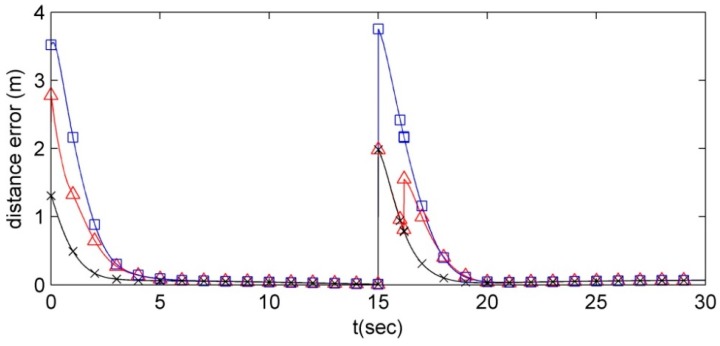
The formation distance errors of three position allocation algorithms (□: fixed-ID matching, ×: optimization, and ∆: proposed CPA).

In addition, to show the performance of the proposed MLC structure and CPA algorithm, they are compared with the rigid allocation and online optimization based methods in Figure 10.

In Figure 10, it can be ensured that whereas the position error of the formation based on the proposed MLC structure and the CPA algorithm is much smaller than the formation with the rigid allocation, the performance of the proposed algorithms remains similar to the optimization based allocation algorithm. The second peak of the position errors of the proposed CPA algorithm after *t* = 15 s in Figure 10 occurs at the competition between the third and the fourth robots due to the limited communication range. From Figure 10, we can hold the advantage that the proposed MLC structure and CPA algorithm provide the similar performance to the optimization algorithm without increasing the cost of the entire system unlike the high cost optimization based allocation algorithm.

Considering the multi-robot system with large number of robots, to show the performance of the proposed MLC structure and CPA algorithm, the following scenario is included. The number of the robots is 100, eleven LCs (e.g., 45th–55th robots) are used, and the vertex set of the formation is *G*{*L*, ψ} where *L* and ψ are as follows:

*L*{1.004(*i* − 50)}, ψ{37π/70} *i* = 1,…, 50 *L*{1.004(*i* − 50)}, ψ{−37π/70} *i* = 51,…, 100

**Figure 11:**
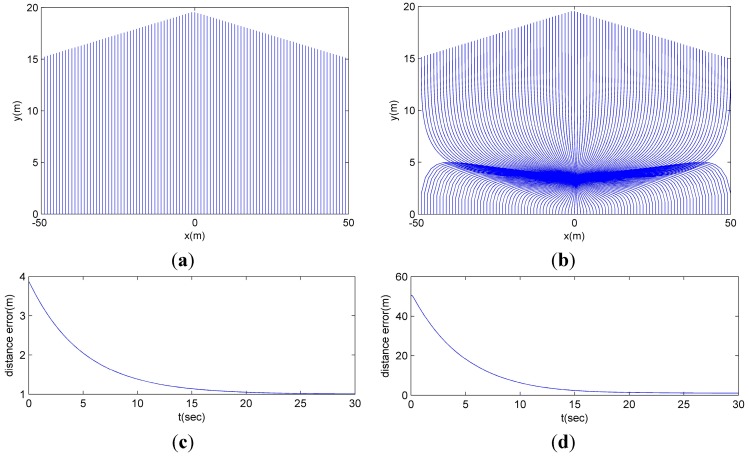
The contrast between the proposed MLC structure and CPA algorithm and the rigid formation. (**a**) The route of the robots employing the proposed MLC structure and CPA algorithm; (**b**) The route of the robots using the rigid formation; (**c**) The distance error of the robots with the proposed MLC structure and CPA algorithm; (**d**) The distance error of the robots using the rigid formation.

The initial conditions of the robots are given as follows: (*x_i_*(0), *y_i_*(0), θ*_i_*(0))*=* (*i* − 50, 0, π/2) for *i* = 1, …, 100. Also, the initial condition and the velocities of the trajectory of the formation are (0, 5.45, π/2) and {*v_r_* = 0.5 m/s, ω*_r_* = 0 rad/s}, respectively. In addition, to compare with the rigid formation, the rigid formation where the *i*th robot tracks the *i*th vertex (*i.e.*, (*x_i_*, *y_i_*, *θ_i_*) → *G*(*i*) = {*L*(*i*), ψ(*i*)}) is considered. Figure 11 shows the results of this scenario with 100 robots.

Figure 11a,c shows the performance of the proposed MLC structure and CPA algorithm and Figure 11b,d shows the performance of the rigid formation. Contrasting the proposed structure and position allocation algorithm and the rigid formation, even though the robots track their desired vertices, the moving cost of the proposed MLC structure and the CPA algorithm is much smaller than the rigid formation.

### 4.2. Experiment Results

**Figure 12:**
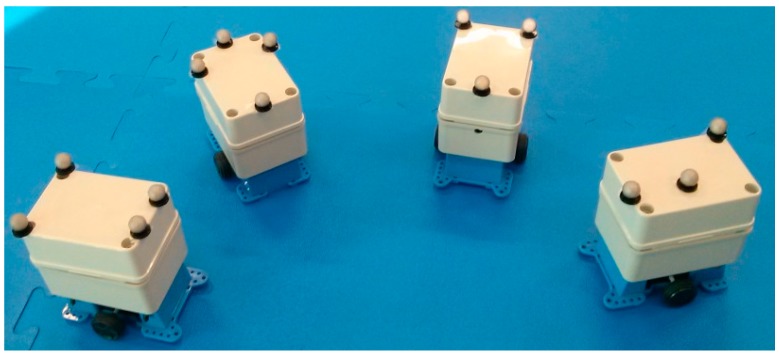
The multi-robot system with four robots

In this paper, the proposed MLC structure and CPA algorithm have been applied to a real multiple robot system which has four small wheeled mobile robots (110 mm × 80 mm × 130 mm, see Figure 12) with differential drive mechanisms.

**Figure 13:**
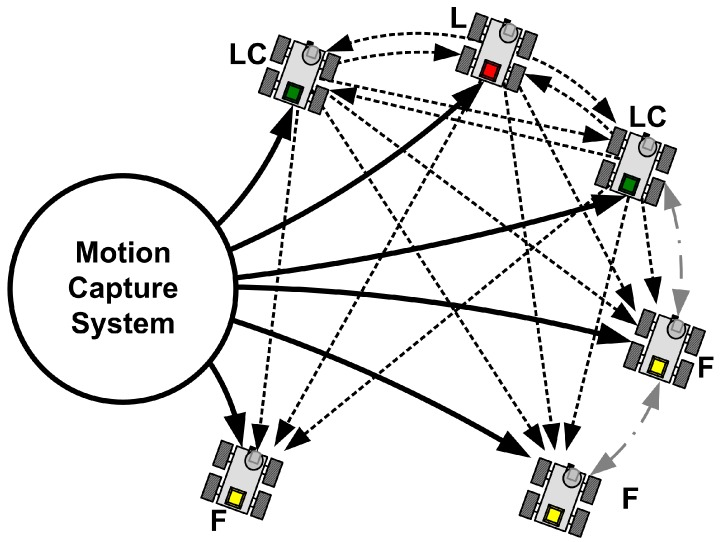
The architecture of the experiment using the motion capture system.

To control the robots, they are equipped with an ATMEGA-128 which is a 16-bit micro-controller, an LM298 which is two-channel motor driver, and a Bluetooth communication module. That is, it is possible that small low cost robots can be employed to realize the proposed MLC structure and CPA algorithm. We assume that each robot can acquire its own positions, thus, the motion capture system [34] is employed to realize the assumption. Even though we use the motion capture system, the experiments maintain the combined network topology and the distributed computation. To do this, each robot acquires its own position from the motion capture system. In addition, the communication network is realized in a server, such that the LCs broadcast their information and the followers communicate with other followers in communication boundary. Figure 13 details the experiment architecture.

In Figure 13, the solid lines indicate the position information from the motion capture system, the dotted lines are information flows from the LCs and the leader, and the dash-dot lines are information flows from the followers. As can be seen in Figure 13, each robot acquires its own position information from the motion capture system, the communication boundary of the follower robots is limited except the LCs. As depicted in Figure 2, to realize the virtual leader structure among the LCs, the communication boundary of the LCs is not limited.

In order to show the implementation results of the proposed algorithms, we include the two scenarios: the leader fault and formation change situations. In these scenarios, the first and second robots are chosen as LCs and the third and fourth robots become the followers. Also, the initial condition of the trajectory of the formation is given as (*x_r_*(0), *y_r_*(0), θ*_r_*(0)) = (0, 0, π/2) and the linear and angular velocities are *v_r_* = 0.3 m/s and ω*_r_* = 0 rad/s, respectively, where *r* notates the reference trajectory.

**Figure 14:**
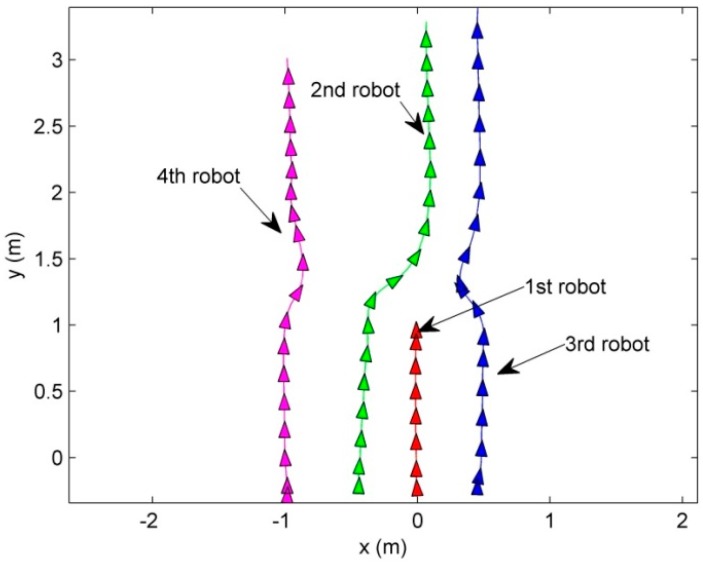
The routes of the robots in the situation of the fault of the leader.

**Figure 15:**
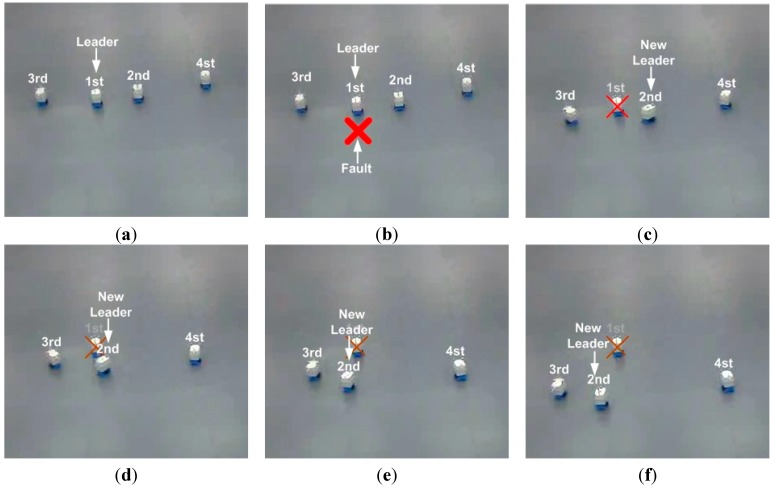
The snapshots of the situation of the fault of the leader.

**Figure 16:**
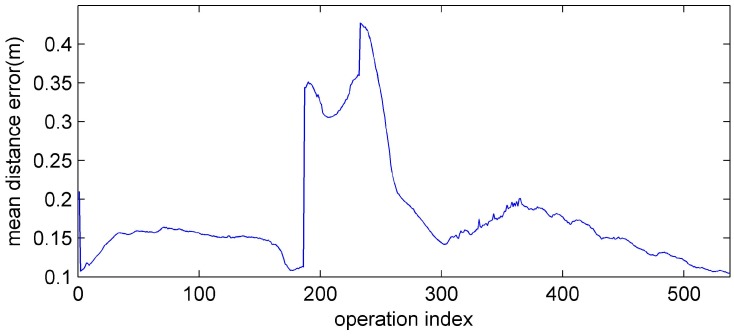
The mean distance error of the robots.

First, to show the performance of the proposed MLC structure and CPA algorithm in the situation of the leader fault, the leader breaks down at the position that the leader passes *y* = 1. The given formation is *G* = {*L*{0, 0.5, 1, 0.5}, ψ{0, π, π , −π}}. The results are presented in Figure 14, Figure 15 and Figure 16. Figure 14 and Figure 15 show the routes of the robots and the snapshots.

As can be seen in Figure 14 and Figure 15, the robots maintain the formation despite of the breakdown of the leader. In Figure 14 and Figure 15, before the leader fault situation, the first robot occupies the leader position and the others move to the desired vertices (see Figure 15a). When the leader breaks down (see Figure 15b), the second robot is chosen as a new leader. The new vertices are generated with respect to the new leader and the followers (the third and fourth robots) are reallocated to the new vertices (see Figure 15b–f). In addition, to show the performance of the control and position allocation, Figure 16 shows the mean distance error of the four robots. 

In Figure 16, before the leader fault situation, the position errors are bounded. When the leader breaks down, the distance error of the formation increases due to the reassignment of the vertices of the formation. After this reassignment of the followers, the distance errors converge to zero and are bounded.

**Figure 17:**
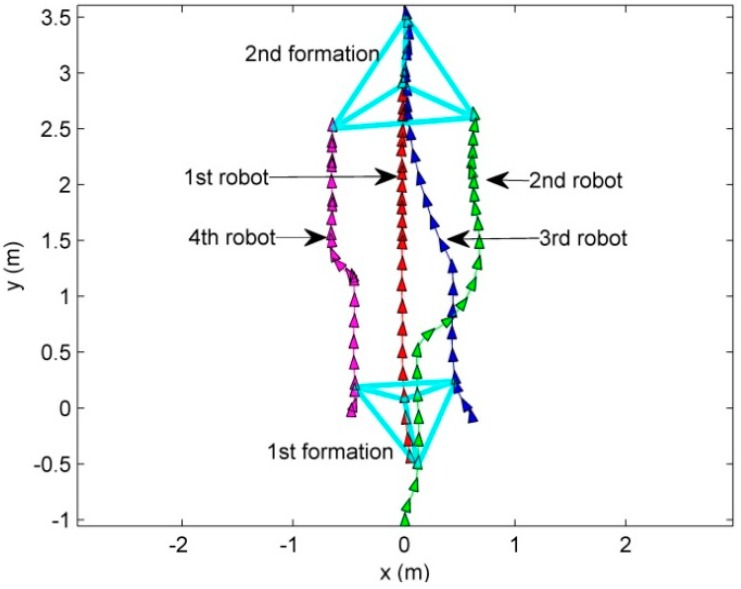
The routes of the robots in the situation of the formation change.

**Figure 18:**
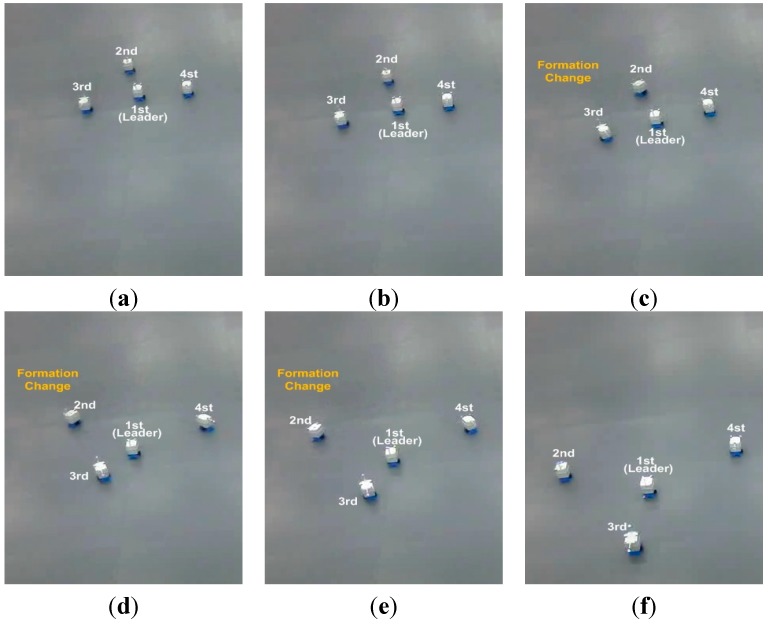
The snapshots of the situation of the formation change.

**Figure 19:**
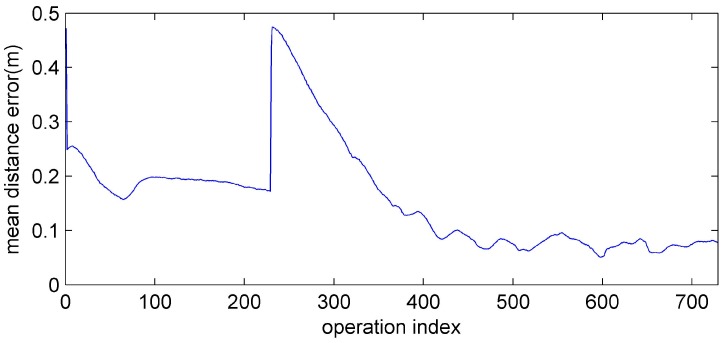
The mean distance error of the robots

The second scenario is the formation change situation where the formation is changed from a Y-shape formation, *G*_1_{*L*_1_{0, 0.5, 0.5, 0.5}, ψ_1_{0, 0, −2π/3, 2π/3}}, to a ∆-shape formation, *G*_2_{*L*_2_{0, 0.707, 0.707, 0.707}, ψ_2_{0, −π/3, π/3, π}}, at the time that the leader passes *y* = 1. Figure 17, Figure 18 and Figure 19 show the performance of the proposed MLC structure and CPA algorithm in the formation change situation. The routes of the robots and snapshots of the second scenario are shown in Figure 17 and Figure 18, respectively. As can be seen in Figure 17 and Figure 18, four robots maintain the given formations without the rigid allocation and the high cost optimization algorithm.

In addition, to show the performance of the control and position allocation, Figure 19 shows the mean distance error of the four robots. In Figure 19, before the formation change, the position errors of the robots are bounded. When the formation shape is changed from *G*1 to *G*2, the distance error of the formation increases since the formation is reconstructed with respect to the changed formation shape. Also, it is ensured that the distance errors converge to zero and are bounded after the reassignment of the followers.

## 5. Conclusions

This paper proposes the multiple leader candidate structure and the competitive position allocation algorithm for formations of multiple robots, which can be applicable for various applications, including environment sensing. By the proposed MLC structure, LC robots replace the lost (or broken) leader using the proposed CPA algorithm. This proposed CPA algorithm allocates the followers to the vertices of the formation using local information. The multi-robot system employing the proposed MLC structure and CPA algorithm achieves robustness against the member robot faults, decentralized position allocation without high cost optimization, and reduction of the moving cost. To ensure the feasibility and performance of the proposed algorithms in real multi-robot systems, we have provided not only simulation results, but also the results of the implementation in a real robotic system.
